# Uniaxially crumpled graphene as a platform for guided myotube formation

**DOI:** 10.1038/s41378-019-0098-6

**Published:** 2019-11-04

**Authors:** Junghoon Kim, Juyoung Leem, Hong Nam Kim, Pilgyu Kang, Jonghyun Choi, Md Farhadul Haque, Daeshik Kang, SungWoo Nam

**Affiliations:** 1 Department of Mechanical Science and Engineering, University of Illinois at Urbana-Champaign, Urbana, IL 61801 USA; 2 Center for BioMicrosystems, Brain Science Institute, Korea Institute of Science and Technology (KIST), Seoul, 02792 Republic of Korea; 3 Division of Bio-Medical Science and Technology, KIST School, Korea University of Science and Technology, Seoul, 02792 Republic of Korea; 4 Department of Mechanical Engineering, George Mason University, Fairfax, VA 22030 USA; 5 Department of Mechanical Engineering, Ajou University, Suwon, 16499 Republic of Korea; 6 Department of Materials Science and Engineering, University of Illinois at Urbana-Champaign, Urbana, IL 61801 USA

**Keywords:** Nanostructures, Nanoscale materials

## Abstract

Graphene, owing to its inherent chemical inertness, biocompatibility, and mechanical flexibility, has great potential in guiding cell behaviors such as adhesion and differentiation. However, due to the two-dimensional (2D) nature of graphene, the microfabrication of graphene into micro/nanoscale patterns has been widely adopted for guiding cellular assembly. In this study, we report crumpled graphene, i.e., monolithically defined graphene with a nanoscale wavy surface texture, as a tissue engineering platform that can efficiently promote aligned C2C12 mouse myoblast cell differentiation. We imparted out-of-plane, nanoscale crumpled morphologies to flat graphene via compressive strain-induced deformation. When C2C12 mouse myoblast cells were seeded on the uniaxially crumpled graphene, not only were the alignment and elongation promoted at a single-cell level but also the differentiation and maturation of myotubes were enhanced compared to that on flat graphene. These results demonstrate the utility of the crumpled graphene platform for tissue engineering and regenerative medicine for skeletal muscle tissues.

## Graphene: crumpled surfaces add a new wrinkle to tissue engineering

A technique that introduces instabilities onto normally flat graphene can enhance the engineering of muscle fibers. Researchers have recently found that applying cyclic electric stimuli to conductive graphene films promotes the growth of tubular myoblast cells that comprise muscle tissue. SungWoo Nam at the University of Illinois at Urbana-Champaign in the United States and colleagues have now developed a ‘crumpled’ form of graphene to further control the morphology of myoblasts. The team transferred graphene film onto a pre-stretched elastomeric tape, and then gradually released the tape’s tension. Microscopy revealed graphene’s transformation into an accordion-like surface featuring nanoscale ridges and valleys. Comparisons with flat graphene showed the new material improved the assembly of myoblasts into longer myotubes by promoting growth along the direction of the crumpled valleys.

## Introduction

Native human tissues possess inherent anatomical directionality, and such directionality endows tissue-specific functions^1^. For example, skeletal muscles have longitudinally aligned myofibrils presenting directional contractile functions in response to stimuli^2^. Ligaments and tendons have unidirectionally aligned collagen fibers and thus absorb sudden mechanical forces by the shearing mechanism of the protein matrix^3^. In addition to those muscular tissues, the hippocampus in brain tissue has directionally sprouting neuronal networks, allowing a unidirectional signal transfer through the CA1-CA3 neural circuit^4^. Because geometrical directionality has a close relationship with function, the recapitulation of anisotropic orientation has been conceived as a key factor in the fabrication of in vitro tissue models^5,6^. One simple yet robust method is the fabrication of anisotropic structures and the seeding of tissue-specific cells on top of tissue scaffolds. Using this method, cells recognize the orientation and surface materials of the underlying substrates and adapt their behaviors based on physicochemical cues. To prepare directional micro/nanostructures, various microfabrication methods, such as photolithography, soft lithography, and electrospinning, have been developed^7–9^.

In the fabrication of in vitro tissue models, the material properties are also an important issue. Skeletal muscle, heart, and brain tissues have electrical stimuli-responsive characteristics. The introduction of cyclic electrical stimuli during the culture period can promote the differentiation and functional maturation of C2C12 mouse myoblast cells^10–12^. Even in the absence of electrical stimuli, simply cultivating C2C12 mouse myoblast cells and neurons on conductive materials enhances differentiation^13^. These results clearly show the importance of the electrically conductive nature of the matrix in the development of electrically active tissue models. In this regard, graphene has substantial potential in the recapitulation of physiological electrical properties because it is a very thin material with high electrical conductivity^14^. Furthermore, graphene shows promise in the regulation of cell functions, such as promoting cell adhesion and enhancing stem cell differentiation^15–17^ because the adhesion of growth factors on a graphene film is promoted by interactions with π–π stacking^18^ and enhanced graphene-plasma membrane interaction, which regulates downstream cell signaling pathways^19^.

To utilize the characteristics and topographical effect of graphene in tissue engineering, a variety of approaches have been introduced. As a straightforward approach, rectangular graphene patterns with a large area of 150 × 1500 μm were fabricated using standard photolithography methods, and myogenic differentiation of C2C12 mouse myoblast cells was demonstrated on the patterns^20^. As a different approach, a suspended graphene sheet was fabricated by transferring a graphene film on top of engineered nanogrooves^21^. On the graphene film, human mesenchymal stem cells showed an aligned morphology due to the nanogrooves beneath the graphene film, and the morphology promoted neurogenic and osteogenic differentiation. The transfer printing of graphene oxide flakes on the substrate allowed the fabrication of strip and grid graphene oxide patterns and demonstrated the graphene-specific adhesion and controlled differentiation of adipose-derived stem cells on the graphene patterned substrates^22^. A graphene-nanofiber hybrid scaffold created by electrospinning polycaprolactone nanofibers on a graphene oxide film was used to explore the potential of this material in oligodendrocyte differentiation^23^. Micrometer-scale graphene oxide wrinkles fabricated by an unconventional fabrication method were shown to be useful in their system in guiding cell alignment^24^. A microgroove-patterned methacrylated gelatin substrate showed enhanced differentiation capabilities compared to a nonpatterned methacrylated gelatin substrate^25^. Moreover, recent research has demonstrated that conductive three-dimensional graphene foam bioscaffolds improve the growth and differentiation of C2C12 myoblasts into functional myotubes^26^. Although previous approaches showed the potential of graphene-based tissue engineering platforms, they usually adapted complex and multistep fabrication techniques for topographically patterned graphene substrates. To utilize novel material properties in tissue engineering applications, a simpler yet robust fabrication method is required.

In this study, we report a tissue engineering platform that can utilize the topographical features and material properties of graphene by inducing mechanical instability in a graphene film. For this purpose, we transferred graphene onto a prestretched elastomer film and subsequently released the stretch so that the film could spontaneously form anisotropic geometrical features via wrinkling phenomena. We then cultured C2C12 mouse myoblast cells on the crumpled graphene and observed myotube formation and maturation in response to the anisotropic geometrical features. On the crumpled graphene substrate, C2C12 myoblasts showed a highly aligned morphology following the wrinkled structures and exhibited enhanced myotube formation compared to those on the flat graphene substrate.

## Results and discussion

### Fabrication and characterization of crumpled graphene

**Fig. 1:**
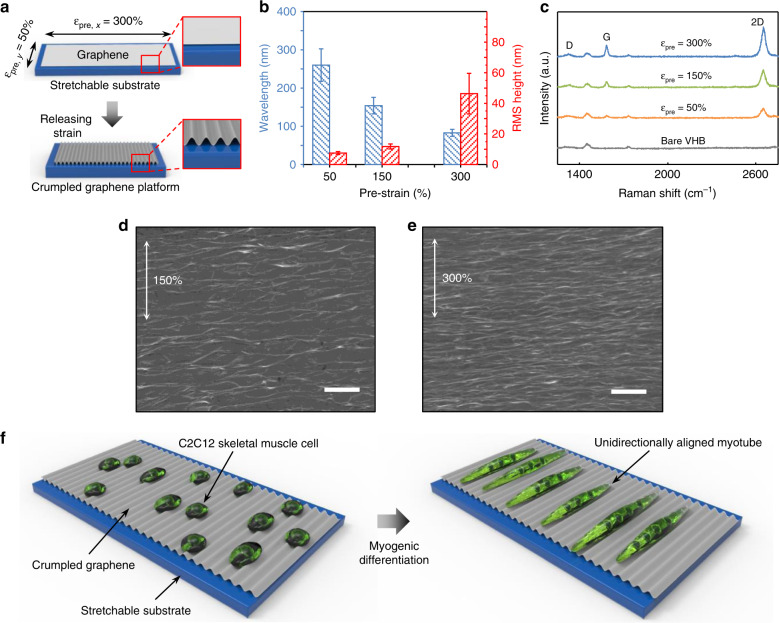
Fabrication and characterization of the anisotropically crumpled graphene platform. **a** Fabrication of the crumpled graphene. By releasing the elastomeric substrate on which a graphene film was attached, unidirectionally crumpled graphene substrates were fabricated. **b** Wavelength and RMS height of crumpled graphene substrates with respect to prestrain. Previous results support that our level of imperfection (i.e., standard deviation of wavelength/height) is less than a length that a cell can recognize^29^. **c** Raman spectroscopy of the crumpled graphene on VHB substrates with varying prestrain and that of the bare VHB film. **d**–**e** Representative SEM images of crumpled graphene fabricated from 150% (**d**) and 300% (**e**) prestrains (scale bars: 1 μm). **f** Schematic illustrations of the myogenic differentiation and alignment of C2C12 cells on crumpled graphene

To fabricate crumpled graphene structures, we transferred chemical vapor deposition (CVD)-grown graphene onto a prestretched elastomeric substrate (VHB tape) using a polydimethylsiloxane (PDMS) block as a temporary stamp. The stretchable substrate was prestretched by different amounts in the *x*-direction and *y*-direction depending on the orientation to induce anisotropic mechanical instability. The major axis was stretched by 150–300%, while the minor axis was stretched by 50% (Fig. 1a). We applied a stretch of 50% in the minor axis direction to compensate for the Poisson tension during the release of the stretchable substrate. By releasing the graphene-stretchable substrate complex, the stiff-soft bilayer structure forms crumpled structures due to the mismatch of the stiffness of graphene (elastic modulus ~1 TPa)^27,28^ and the stretchable substrate (elastic modulus ~0.59 MPa, Fig. S1) (Fig. 1a). As shown in Fig. 1 and S2–S4, the graphene spontaneously formed an anisotropic, nanoscale, wave-like structure without notable mechanical fractures. The k-vector of the crumpled graphene was parallel to the major axis direction. The wavelength of the crumpled graphene decreased as the prestrain increased (i.e., *ca*. 260 nm, 150 nm, and 80 nm for 50, 150, and 300% prestrain, respectively), whereas the amplitude (displayed as the root mean square (RMS) height) increased in response to increased prestrain (i.e., *ca*. 8 nm, 12 nm, and 46 nm for 50, 150, and 300% prestrain, respectively) (Fig. 1b; Table S1 and S2). Representative scanning electron microscopy (SEM) images show the structures of crumpled graphene in the cases of 150 and 300% prestrain (Fig. 1d, e). Raman spectroscopy further revealed that the crumpling did not induce any permanent damage to the graphene film; the D band (~1350 cm^−1^) intensity remained constant as the prestrain increased (Fig. 1c). Furthermore, the intensities of the Raman peaks from graphene increased as the prestrain increased, which indicates that the graphene wrinkles were densified with a larger prestrain.

### Differentiation of skeletal muscle cells

**Fig. 2:**
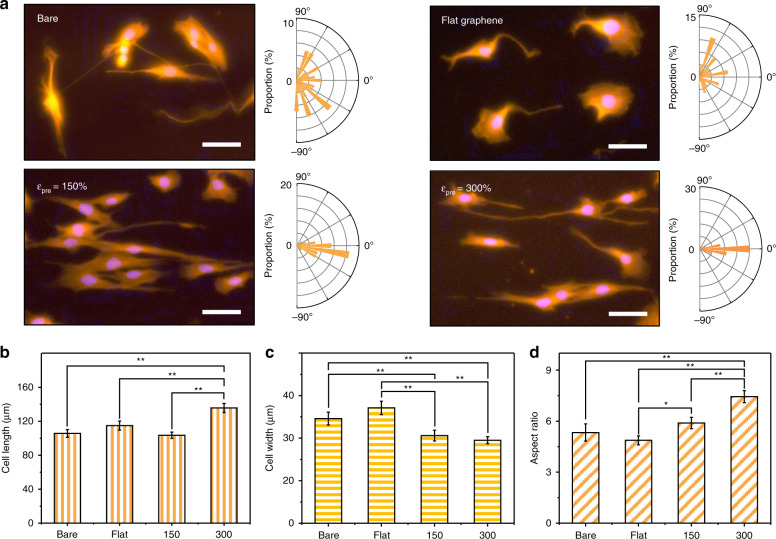
Morphology of single-cell level C2C12 cells 3 days after seeding on the anisotropically crumpled graphene platform. **a** Representative immunofluorescence images of C2C12 cells on bare (no graphene), flat (with graphene), and 150 and 300% prestrained crumpled graphene platform (Scale bar: 50 μm). The magnification of Fig. 2a was 20×. For visualization, C2C12 mouse myoblast cells were stained with TRITC-conjugated phalloidin. Red: F-actin (phalloidin), blue: nuclei (DAPI). The angular distribution of cells was quantified and visualized with the orientation rose plot. **b**–**d** Morphology analysis of C2C12 cells 3 days after seeding: (**b**) cell length, (**c**) cell width, and (**d**) aspect ratio (ratio of long axis to short axis). Significance: **p < 0.01 and *p < 0.05. Data are represented as the mean ± SE (*n* = 5, in the case of the myotube aspect ratio, *n* = 100)

To demonstrate the utility of crumpled graphene in skeletal muscle tissue engineering, C2C12 mouse myoblast cells were seeded on various crumpled graphene platforms (Fig. 1f). Here, the flat graphene sample was used to identify the contribution of topographical cues to the C2C12 mouse myoblast cell behaviors. Three days after seeding, the cell morphologies and their alignment behaviors were quantified using fluorescence microscopy (Fig. 2). As shown in Fig. 2a, on the flat (with graphene) substrates, the C2C12 cells showed a random orientation due to the absence of topographical guidance. However, the cells showed preferential alignment, with spindle-shaped morphologies, along with a crumpled topography in the case of the 150 and 300% prestrain platforms. Such alignment phenomena of C2C12 coincided with those observed in previous studies^13,29–32^, showing contact guidance in response to the underlying surface topography.

To compare the differences in those cell alignment behaviors, we conducted a statistical analysis (Table 1). The circular standard deviation (the standard deviation of the cell orientation angles) decreased as the prestrain increased from 150 to 300%, indicating stronger topography-induced guidance effects with higher prestrain. Rao’s spacing test of uniformity is generally used to demonstrate whether the angular distributions of data are significantly different among experimental conditions. A sufficiently small *p*-value indicates that the angular distribution is significantly different from the ‘uniform’ distribution, with given statistical reliabilities corresponding to the *p*-values^33^. In the 150 and 300% prestrain cases, *p*-values smaller than 0.01 indicate a significantly different distribution of angle values from the uniform distribution. The Mardia–Watson–Wheeler test was further used to confirm the equality of the two angular populations^34^. A sufficiently small *p*-value indicates that the angular populations of two data sets are significantly different with *p*-value-specific statistical reliabilities. The Mardia–Watson–Wheeler test between the data sets, such as flat:150% and flat:300%, showed significantly small p-values, indicating a significant difference between the cases in terms of the angular populations. These statistical analysis results consistently showed the utility of the anisotropically crumpled graphene platform for the unidirectional alignment of C2C12 cells.

**Table 1:**
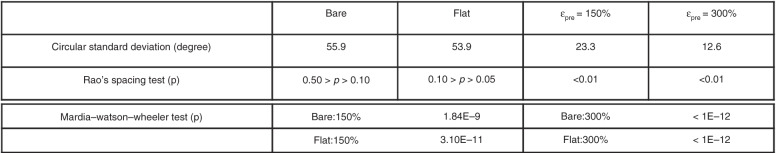
The statistical analysis of the circular standard deviation, Rao’s spacing test of uniformity, and Mardia–Watson–Wheeler test for single cell-level C2C12 cells

The morphological indices, including the cell length, cell width, and aspect ratio, were different depending on the various platforms (Fig. 2b-d). For example, C2C12 mouse myoblast cells on the 300% prestrain samples showed a much longer cell length than the other cases. Conversely, the cells on the 150 and 300% prestrain samples displayed narrower cell widths than those on the bare and flat substrates. Due to the increased cell length and decreased cell width, the cells on the 150 and 300% prestrain samples showed higher aspect ratios (the ratio of the long axis to the short axis), and the aspect ratio value was even larger in the 300% case than in the 150% case. The longer cell length and higher aspect ratio in the case of the 300% prestrain samples correlated with the amplitude of the crumpled graphene (Fig. 1b). Although the wavelength of the crumpled graphene decreased as the prestrain increased, the amplitude increased as the prestrain increased (Fig. 1b). This correlation between the cell morphological indices and the graphene wrinkle amplitude indicates the importance of topographical height in the guidance of cell shape and alignment. Previous studies reported that topography-induced alignment was more effective with nanogrooves with larger height values^35,36^. In this regard, the higher amplitude in the case of 300% prestrain is more beneficial than that for 150% prestrain, and thus, cell alignment and elongation were promoted in the 300% prestrain case.

**Fig. 3:**
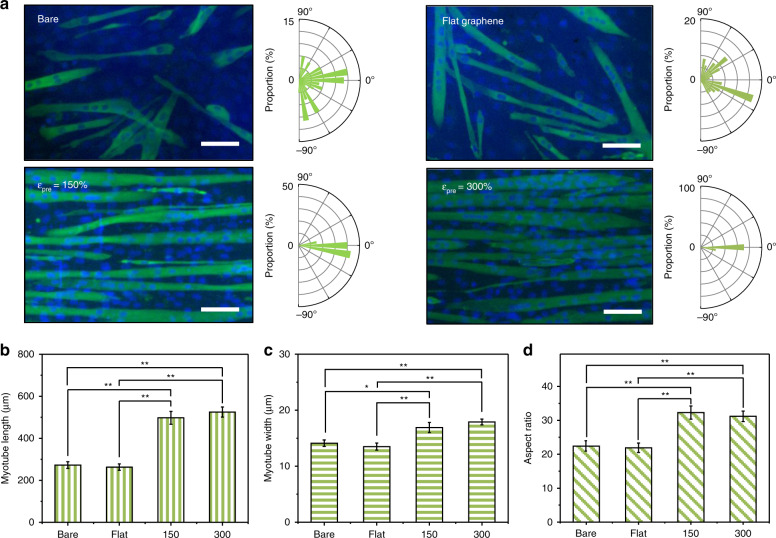
Morphology of myotubes on crumpled graphene. **a** Representative fluorescence images and angular distribution of the myotubes on anisotropically crumpled graphene. After 7 days of differentiation of C2C12 cells in differential media, myotubes and nuclei were stained for anti-myosin heavy chain (green) and DAPI (blue), respectively (scale bar: 100 μm). The magnification of Fig. 3a was 10×. **b**–**d** Quantitative analysis of myotube morphologies: (**b**) myotube length, (**c**) myotube width, and (**d**) aspect ratio. Significance: **p < 0.01 and *p < 0.05. Data are represented as the mean ± SE (*n* = 5, in the case of the myotube aspect ratio, *n* = 100)

To demonstrate the role of the crumpled graphene platform in the induction of myogenic differentiation of C2C12 mouse myoblast cells, the cells were induced to be differentiated in low-serum media for 7 days. The C2C12 cells fused and subsequently formed myotubes (Fig. 3 and S5). The myotubes on the 150 and 300% prestrain samples showed clear alignment along the crumpled orientation of the graphene film (Fig. 3a). The alignment pattern of myotubes on various samples was also quantified using statistical analysis methods (Table 2). The circular standard deviation values were significantly lower for the 150 and 300% prestrain cases than for the flat case, and the values decreased as the prestrain value increased from 150 to 300%, which is similar to the individual C2C12 cell results. Rao’s spacing test of uniformity showed significantly different alignment patterns of cells on the 150 and 300% samples (*p* < 0.01). The Mardia–Watson–Wheeler test demonstrated significant differences between the angular populations on various samples, such as flat:150% and flat:300% (*p* < 1e^−12^). The morphological indices of myotubes were also quantified. The length, width, and aspect ratio of myotubes were larger in the case of the crumpled graphene substrates than the flat substrates (Fig. 3b-d). However, there were no notable differences between the 150 and 300% prestrain cases.

**Table 2:**
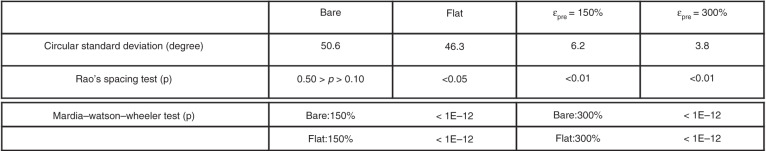
The statistical analysis of the circular standard deviation, Rao’s spacing test of uniformity, and Mardia–Watson–Wheeler test for myogenic differentiation of C2C12 cells

**Fig. 4:**
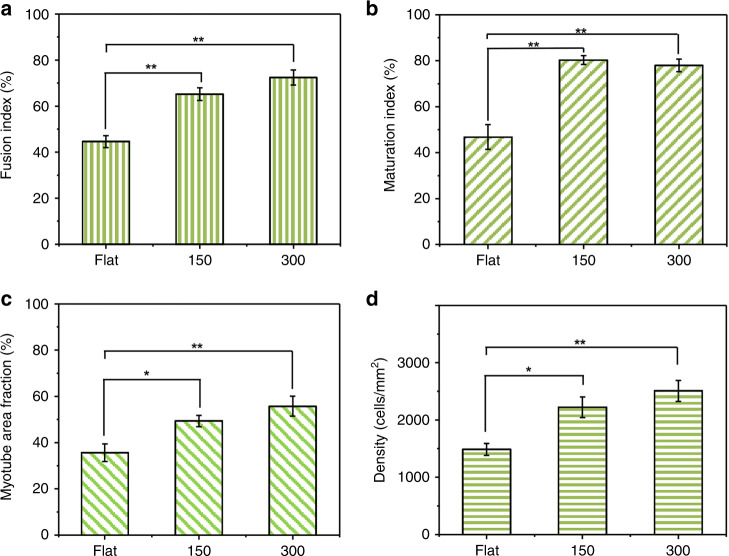
Quantified maturation indices of myotubes on the anisotropically crumpled graphene platforms. **a** Fusion index. **b** Maturation index. **c** Myotube area fraction. **d** Density of cells on the flat/crumpled graphene. Significance: **p < 0.01 and *p < 0.05. Data are represented as the mean ± SE (*n* = 5, in the case of the myotube aspect ratio, *n* = 100)

The myogenic differentiation efficiency was evaluated with the fusion index, maturation index, myotube fraction, and myotube density (Fig. 4). The fusion index denotes the ratio of the nuclei number in myocytes with multiple nuclei, and the maturation index is the proportion of myotubes having five or more nuclei in a single myotube. The myotube area fraction is the fraction of area covered by myotubes. The density was defined as the number of nuclei within the unit area (mm^2^). These four indices were higher for crumpled graphene substrates (150 and 300%) than for flat substrates. Similar to the results of the morphological indices of the myotubes, there were no significant differences in those four indices between the 150 and 300% prestrain cases. According to previous studies, neither too large (>20 μm)^37^ nor too small (<70–80 nm)^29^ size patterns affect myotube fusion, implying the limited effect of feature size control over C2C12 differentiation.

To examine the change in stiffness with respect to the applied prestrain and ultimately to the cultured cell behaviors on the crumpled graphene substrates, we measured the elastic modulus of the crumpled graphene using a nanoindenter (Fig. S1). As the prestrain increased from flat to 300%, the stiffness increased from 0.628 MPa to 1.64 MPa. However, in between the 150 and 300% prestrain cases, the increase in stiffness was small from 1.43 MPa to 1.64 MPa for the 150 and 300% prestrain cases, respectively. Although some reports indicated that the increased stiffness of nanogrooves induced further elongation of cells along the pattern orientation^38^, such stiffness-dependent elongation was not observed in our study, presumably because of the small difference in stiffness.

From the quantification, we found that at the single C2C12 mouse myoblast cell level, crumpled graphene with a uniaxial topography increases the cell length while decreasing the width. Furthermore, as the prestrain increases from 150 to 300%, the length and aspect ratio of the individual cells tend to increase due to the increased height of the surface texture. However, after myotube formation (i.e., myogenic differentiation), such morphological differences between the 150 and 300% prestrain samples decreased.

Furthermore, C2C12 mouse myoblast cells and differentiated myotubes on bare VHB and flat graphene on VHB (Figs. 2–4) do not show significant differences in terms of their morphological indices. We believe that the similarity of the mechanical properties of the substrates mainly guided their similarities. Previous studies indicate that the morphology of the cultured cells is mainly controlled by the mechanical properties of the substrates, rather than the chemical moieties of the substrates^39,40^. As reported in previous studies^41^, an ultrathin membrane of a rigid material has a quite limited effect on the overall mechanical properties when layered on a soft substrate. Therefore, we believe that the similarities in the mechanical properties of VHB and graphene on VHB resulted in marginal differences in the morphological indices of C2C12 mouse myoblast cells before and after differentiation.

Our crumpled graphene platform is a significant departure from previous approaches for the following reasons. First, the geometrical size (wavelength and amplitude) of our platform is much smaller than that of the platforms used in previous studies, and our platform thus can stimulate cell behaviors and functions more precisely. For example, previous studies fabricated wrinkled graphene oxide films with a wavelength and amplitude of a few micrometers to a few tens of micrometers due to the thick graphene oxide film^31^. However, in this study, we fabricated a nanoscale (wavelength of *ca*. 80 nm and 150 nm; and RMS height of *ca*. 46 nm and 12 nm; for 300 and 150% prestrain, respectively) topography using a CVD-grown monolayer graphene. Although, as shown in Fig. 1b, our crumpled graphene structures are not perfectly ordered (1-standard deviation <50 nm) compared to patterns fabricated by photolithography techniques, individual C2C12 cells and differentiated myotubes displayed clear alignment and elongation in the crumpled orientation. This clear contact guidance of C2C12 cells on the crumpled graphene substrate and the previous result in which cells might not recognize a sufficiently small feature size (<70–80 nm)^29^ support that the imperfections of crumpled graphene have a negligible effect on cell behaviors. The micron-scale topography is known to have the ability to align cells towards a topographical orientation but not to effectively guide cell differentiation because the feature size is much larger than that of focal adhesions^42^. Conversely, the nanoscale topography can control both alignment and differentiation because the sizes of the features and focal adhesions are comparable^43^. Second, although previous studies reported the superior properties of graphene in the myogenic differentiation of C2C12 cells^20^, the synergistic role of the surface topography and the material properties of graphene have not been clearly demonstrated.

Although the differentiation of C2C12 mouse myoblast cells into myotubes is mainly discussed in this study, the proliferation and metabolism of cells will be important in future studies for the application of our crumpled graphene platform in skeletal muscle tissue engineering. According to previous studies, the morphological indices of differentiated myotubes are closely related to the gene expression of mature myogenic markers^6,13,44^. Such morphology-controlled cell behaviors are also commonly observed in other cell types, such as immune cells and human mesenchymal stem cells^45,46^. Therefore, we believe, although further in-depth studies are needed, that the morphology control can control the differentiation and maturation of mouse myotube cells. As reported in previous studies, culturing mouse myoblast cells (especially C2C12 cells) on a nanotopography upregulates multiple genes related to myogenic differentiation, such as Myf5, MyoD, and MyoG. The enhanced gene expression of myogenic differentiation was evident irrespective of the surface characteristics, including the gelatin-coated poly(lactic-coglycolic acid) (PLGA)^6^, sphingosine-1-phosphate-conjugated PLGA^47^, and gold-coated polyurethane acrylate (PUA) substrates^13^. In addition, microgroove patterns also upregulated the expression levels of focal adhesion-related, actin cytoskeleton-related, and MAPK signaling-related genes^48^. Furthermore, on multiscale micro-patterns and nano-patterns, aligned nanofibers on top of microgrooves with orientations perpendicular to each other, C2C12 cells preferentially aligned following the nanofibers rather than the microgrooves^49^. These results indicate, at least in adhesion and alignment aspects, C2C12 cells prefer nanoscale patterns over microscale patterns. Based on these earlier findings, future investigations on the upregulation of various myogenic genes induced by the crumpled graphene substrate will provide further evidence of the impact of the crumpled topography on myogenic differentiation. The observation of cross-striation will also provide evidence of myotube maturation. In addition to gene expression, the effect of extracellular matrix components on differentiation and subsequent production by the cells can be studied in the future. Previous researchers reported that the laminin protein significantly promotes cell adhesion and differentiation of C2C12 cells^26,50,51^. Furthermore, we expect that these electrically active, nanotopographic crumpled graphene substrates can electrically stimulate cells. According to previous studies, consistent electrical stimulation can enhance the differentiation and maturation of skeletal muscle cells^25,26,52–54^. Although, in this work, there was no significant difference in the experimental results between ‘bare’ and ‘flat’ substrates due to the absence of electrical stimulation and the similarity of the elastic moduli (Fig. S1), an evaluation of the effect of electrical stimulation on crumpled graphene would be meaningful in the future^52^. Finally, human-originating skeletal cells can be used to validate the applicability of crumpled graphene platforms for skeletal muscle tissue engineering. We believe that future studies on the above aspects may further strengthen the usefulness of crumpled graphene platforms for skeletal muscle tissue engineering.

## Conclusions

In this study, we report crumpled graphene, a monolithically defined graphene with a nanoscale wavy surface texture, as a tissue engineering platform that can efficiently promote aligned C2C12 mouse myoblast cell differentiation. By releasing the anisotropic prestrain of an elastomer-graphene bilayer structure, unidirectionally crumpled graphene substrates were fabricated. When C2C12 mouse myoblast cells were seeded, they showed an elongated and aligned morphology in response to the increase in prestrain. After myogenic differentiation, the myotubes had a more elongated morphology and showed better maturation on the crumpled graphene substrates than on the flat substrates. These results showed that crumpled graphene substrates with unidirectional topography can be used as an effective platform for skeletal muscle tissue engineering in terms of alignment, morphology, and maturation. Furthermore, given the simplicity of crumpled substrate fabrication and the simultaneous electrical sensing/stimulation capability of graphene, we believe that our monolithic crumpled graphene platform could allow future innovations in skeletal muscle tissue engineering.

## Materials and methods

### Fabrication of the crumpled graphene structure on a flexible substrate

The crumpled graphene structure was fabricated by modifying the protocols of previous studies^55,56^. Graphene was synthesized on a 25 μm-thick copper (Cu) foil (Alfa Aesar, MA) by a chemical vapor deposition (CVD) system (Rocky Mountain Vacuum Tech Inc., CO). Under a hydrogen (H_2_) gas environment (50 sccm) at 150 mTorr, the chamber was heated to 1050 °C. After annealing at 1050 °C for 2 h, methane (CH_4_) and H_2_ gases (CH_4_: 100 sccm and H_2_: 50 sccm) were introduced into the chamber at 520 mTorr for 2 min. Then, the chamber was cooled down to room temperature slowly under an argon (Ar) gas environment (500 sccm) at 330 mTorr. After the synthesis of graphene, a polydimethylsiloxane (PDMS) block was attached on the graphene side of the Cu foil, and then Cu was etched with 1 M FeCl_3_ solution. For a flexible substrate, a very high bond (VHB) substrate (3 M VHB 4910, *t* = 1.143 mm) was used. After stretching the VHB substrate in biaxial directions, with a larger prestrain applied to one direction (e.g., 300% in the *x* direction and 50% in the *y* direction; *ε*_pre*, x*_ = 300% and *ε*_pre*, y*_ = 50%) to induce uniaxial deformation, the graphene on the PDMS block was transferred onto the stretched VHB substrate. Due to the mechanical limitation of the VHB substrate, the stretching of the VHB substrate was controlled to be less than 400%. After transfer, the stretched VHB substrate was released, and the crumpled graphene structure was obtained. The surface structure of the crumpled graphene was monitored with a scanning electron microscope (SEM, S-4800, Hitachi) and an atomic force microscope (AFM, MFP-3D, Asylum Research), and the integrities of the crumpled graphene at various tensile strain conditions between 0 and 300% were evaluated with a Raman spectroscope (micro PL/Raman microscope system, Renishaw) using a 633 nm wavelength laser. Nanoindentation was carried out with the same AFM system used for imaging.

### Cell morphology and angular orientation analysis

Three types of cell substrates were prepared: two crumpled graphene substrates (*ε*_pre, *x*_: 300 and 150%) and a flat graphene substrate (50% prestrain). The prepared substrates were sterilized with alcohol and ultraviolet (UV) light. Then, mouse skeletal myoblast C2C12 cells were seeded at a density of 5000 cells/cm^2^ on sterilized graphene/VHB substrates. The cells were maintained in growth media (GM, DMEM containing 10% FBS and 1% penicillin-streptomycin) in a humidified atmosphere with 5% CO_2_ at 37 °C. The GM was replaced every 24 h. After 3 days of cell culture, the morphology and angular orientation data of the cells were analyzed using cytoskeleton staining. The cells were fixed with 4% paraformaldehyde, permeabilized with 0.1% Triton X-100, and stained with TRITC-phalloidin (Sigma-Aldrich, USA) and DAPI (Thermo Fisher Scientific, USA). The actin filaments and nuclei were observed using a fluorescence microscope (IX81, Olympus). From the obtained fluorescence images, cell width, length, aspect ratio, and angular orientation data were analyzed with ImageJ software. We analyzed 5 different samples from four kinds of cell substrates and monitored over 100 cells from each sample. We conducted Rao’s spacing test to evaluate the uniform distribution of angular orientation data of cells and the Mardia–Watson–Wheeler test to evaluate the identical distribution of the angular orientation data of cells.

### Myogenic differentiation and alignment analysis

Two types of crumpled graphene substrates (*ε*_pre*, x*_: 300 and 150%) and a flat graphene substrate were prepared and sterilized with alcohol and UV. Mouse skeletal myoblast C2C12 cells were seeded at a density of 5000 cells/cm^2^ on sterilized graphene/VHB substrates. The seeded cells were grown in GM at 37 °C and 5% CO_2_. GM was replaced every 24 h. When the cells covered 80% of the substrate, GM was replaced with differential media (DM, DMEM containing 2% horse serum and 1% penicillin-streptomycin). The cells were incubated at 37 °C and 5% CO_2_, and the DM was replaced every 12 h. After 7 days of differentiation, cells were fixed, permeabilized and blocked with 4% paraformaldehyde, 0.1% Triton X-100 and 1% bovine serum albumin. For immunostaining, cells were incubated with the primary antibody MF20 (anti-myosin heavy chain (MHC)), Developmental Studies Hybridoma Bank ((DSHB), University of Iowa) overnight at 4 °C, followed by incubation with the secondary antibody (fluorescein isothiocyanate (FITC)-conjugated goat anti-mouse IgG, Thermo Fisher Scientific) and DAPI (Thermo Fisher Scientific) for 2 h at 37 °C. Myotube formation and alignment were observed using a fluorescence microscope (IX81, Olympus) and analyzed with ImageJ software. We analyzed 5 different samples from four kinds of cell substrates and monitored over 100 myotubes from each sample. To evaluate the uniform distribution and identical distribution of myotube alignment data, Rao’s spacing and Mardia–Watson–Wheeler tests were conducted. To evaluate the myogenic differentiation efficiency, we analyzed the fusion index (the ratio of the nuclei number in myocytes with two or more nuclei), maturation index (the percentage of myotubes having five or more nuclei), myotube area fraction and cell density from each sample.

### Statistical analysis

All of the quantitative data are expressed as the mean ± standard error of the mean. Statistical analysis was performed by means of one-way analysis of variance. For the determination of statistical significance, *p*-values smaller than 0.05 were considered to be significant.

## Supplementary information

Supplementary Information
