# Supplementary material for: Uniaxially crumpled graphene as a platform for guided myotube formation
Source: Microsyst Nanoeng. 2019 Nov 4;5:53. doi: 10.1038/s41378-019-0098-6 (PMC6826050; doi:10.1038/s41378-019-0098-6)
Supplement: Supplementary file 1 — Supplementary Information [file 41378_2019_98_MOESM1_ESM.docx]

Supplementary Information for

**Uniaxially Crumpled Graphene as a Platform for Guided Myotube Formation**

Junghoon Kim,^1†^ Juyoung Leem,^1†^ Hong Nam Kim,^2,3†^ Pilgyu Kang,^1,4^ Jonghyun Choi,^1^ Md Farhadul Haque,^1^ Daeshik Kang,^5,*^ and SungWoo Nam^1,6*^

*^1^ Department of Mechanical Science and Engineering, University of Illinois at Urbana-Champaign, Urbana, IL 61801, United States*

*^2^ Center for BioMicrosystems, Brain Science Institute, Korea Institute of Science and Technology (KIST), Seoul 02792, Republic of Korea*

*^3^ Division of Bio-Medical Science & Technology, KIST School, Korea University of Science and Technology, Seoul 02792, Republic of Korea*

*^4^ Department of Mechanical Engineering, George Mason University, Fairfax, VA 22030, United States*

*^5^ Department of Mechanical Engineering, Ajou University, Suwon 16499, Republic of Korea*

*^6^ Department of Materials Science and Engineering, University of Illinois at Urbana-Champaign, Urbana, IL 61801, United States*

*^†^These authors contributed equally.*

*Correspondence:

Prof. SungWoo Nam

email: [swnam@illinois.edu](mailto:swnam@illinois.edu)

Phone: +1-217-300-0267

Prof. Daeshik Kang

email: [dskang@ajou.ac.kr](mailto:dskang@ajou.ac.kr)

Phone: +82-31-219-2345

**Biocompatibility and Toxicity**

In spite of the wide use of graphene and graphene-related materials in tissue engineering, there are concerns on the biocompatibility of graphene substrates. For instance, the previous studies have indicated that the graphene and graphene-related substrates may cause various side effects. These side effects include (1) increased reactive oxygen species level, (2) trigger of apoptosis, and (3) increased DNA damage.^1^ The toxicity of graphene and graphene-related materials such as graphene oxide and reduced graphene oxide has been reported previously.^1,2^ However, in the case of skeletal muscle tissue, the accumulation of graphene-related materials in the skeletal muscle tissue is quite limited (finding ratio of ~1% in muscle)^3^, and the exposure of C2C12 mouse myoblast cells in a high dose of 1.5 mg/mL for 24 hours did not cause any notable toxicity.^4^ Therefore, although further investigation is required, we believe that graphene and graphene-related materials do not cause significant toxicity for the skeletal muscle tissue engineering. Meanwhile, according to the Regulatory Data Sheet provided by the vendor (3M Inc.), VHB substrate is composed of ‘nonconductive’ acrylate polymer, and it is biocompatible and nontoxic.^5^

**Delivery Methods**

For the skeletal muscle tissue engineering, the delivery methods of cultured skeletal muscle construct to the damaged site is an important issue. The injection of trypsinized single cells in a high density is a simple approach, but the loss of injected cells, immune rejection, and poor engraftment of cells have been considered as limitations.^6^ To address these viability issues, encapsulation of viable cells using biocompatible materials such as hydrogels has been proposed and demonstrated the enhanced cell viability after the in vivo injection.^7,8^ The transplantation of cultured cells in a sheet form with the substrates is arising as an efficient way for tissue regeneration, especially for the tissues with inherent structural directionality such as skeletal muscle.^9^ Considering that the crumpled graphene platform can guide unidirectional cell alignment, the engraftment of differentiated myotubes in a cell sheet form may promote rapid regeneration of damaged skeletal muscle tissues.


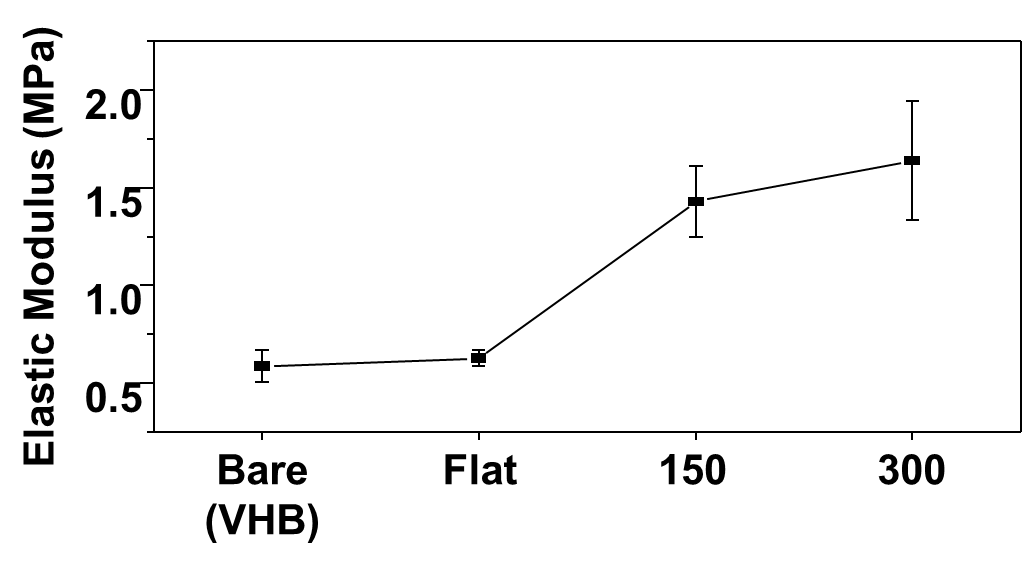


**Fig. S1** Elastic modulus of crumpled graphene substrates with respect to applied prestrain.


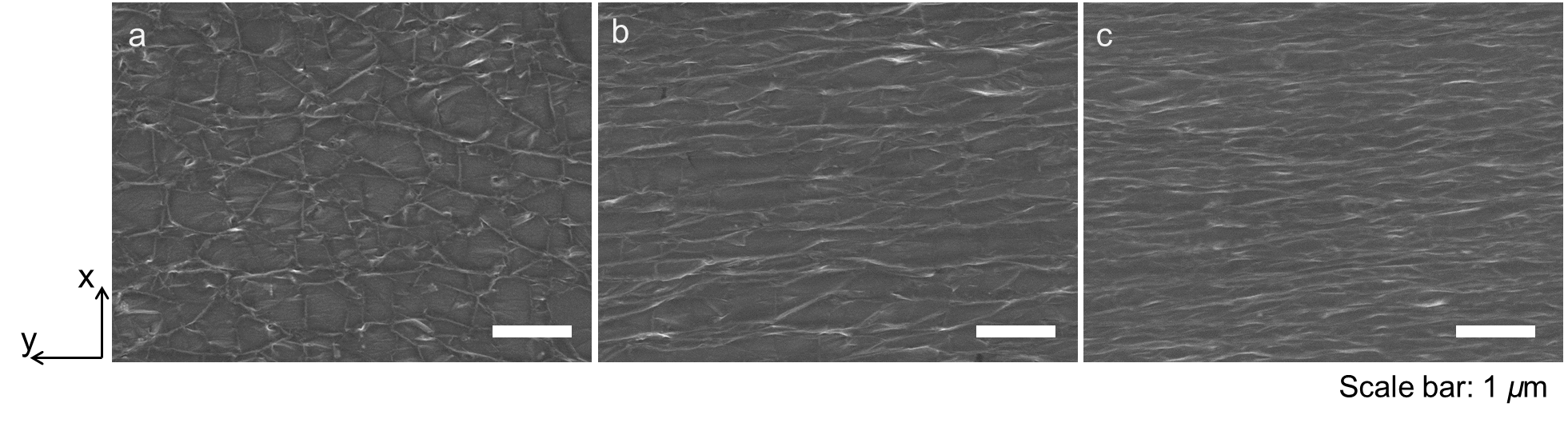


**Fig. S2** SEM images to show topography difference between (a) flat graphene sample prepared with prestrains of 50% in x-axis and 50% in y-axis and (b, c) crumpled samples. Images in panels (b) and (c) are presented in Fig. 1 and used here to show the topographical difference.


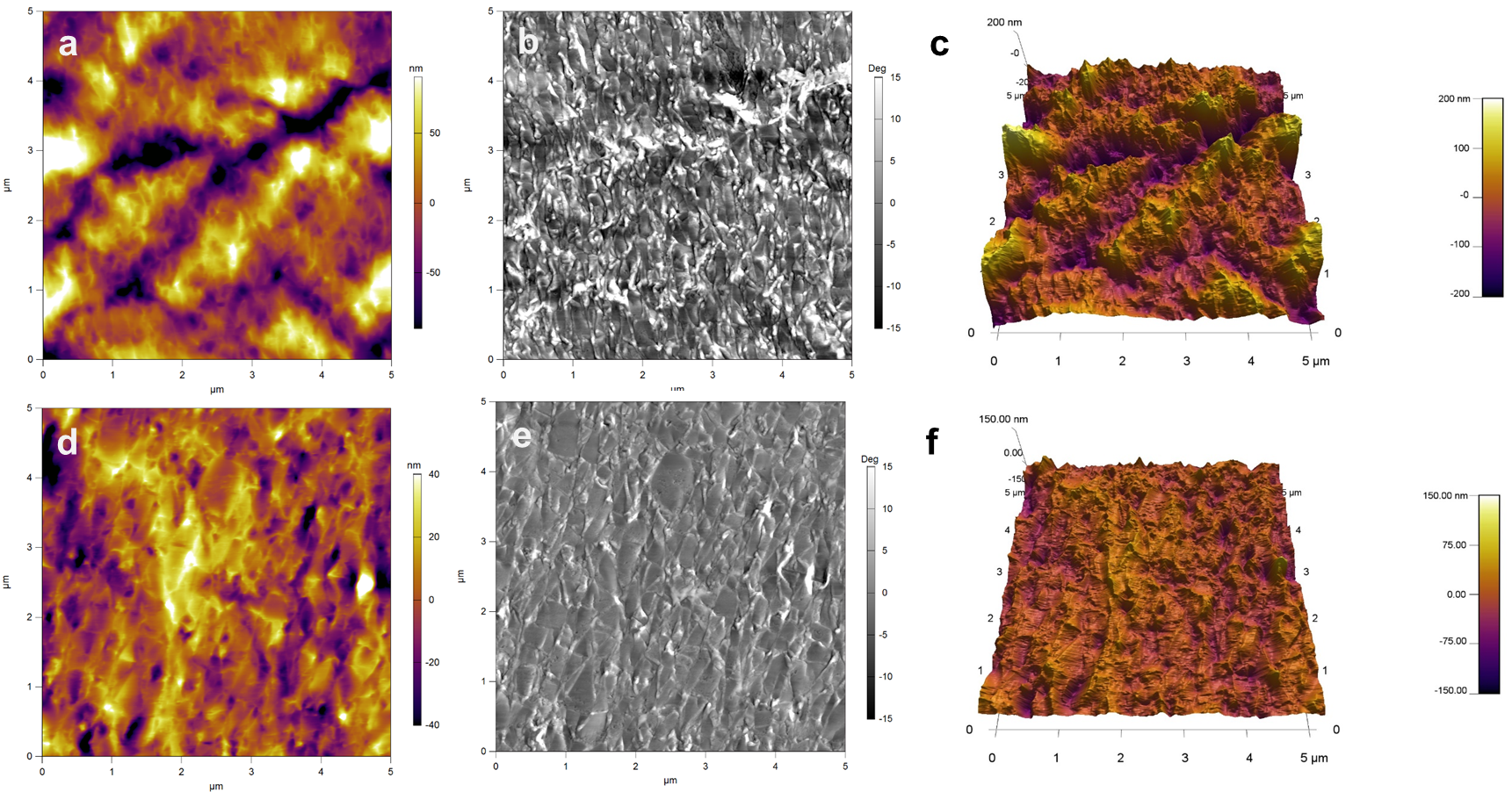


**Fig. S3** AFM results of uniaxially crumpled graphene structures fabricated with 300% and 150% prestrains. (a) Topography, (b) phase image, and (c) three-dimensional topography of 300% sample. (d) Topography, (e) phase image, and (f) 3D topography of 150% sample.


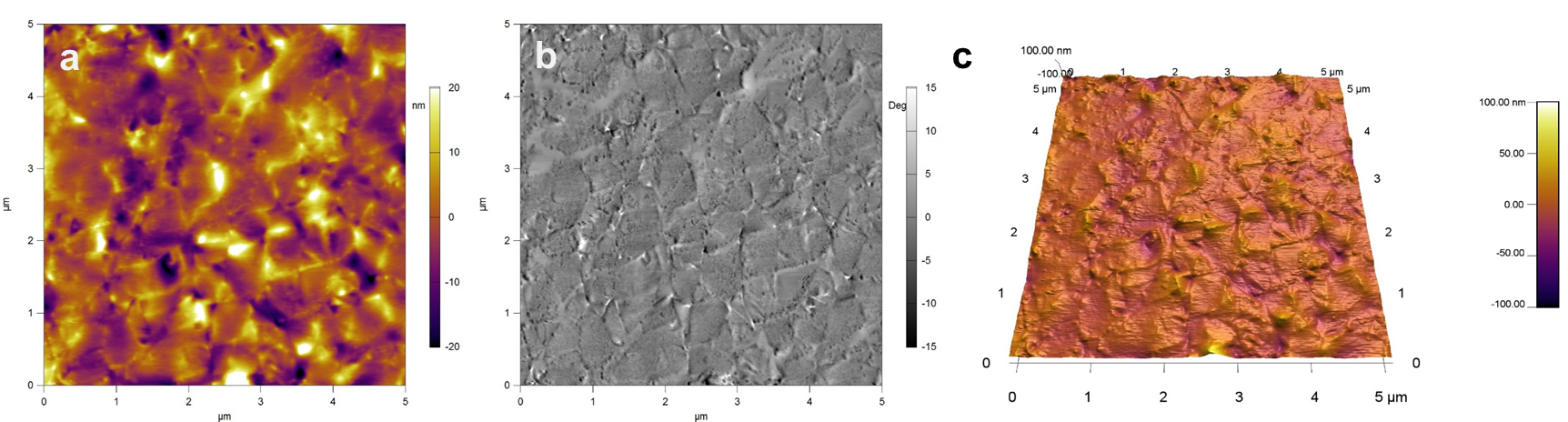


**Fig. S4** AFM results of a flat graphene sample: (a) topography, (b) phase, and (c) 3D topography.


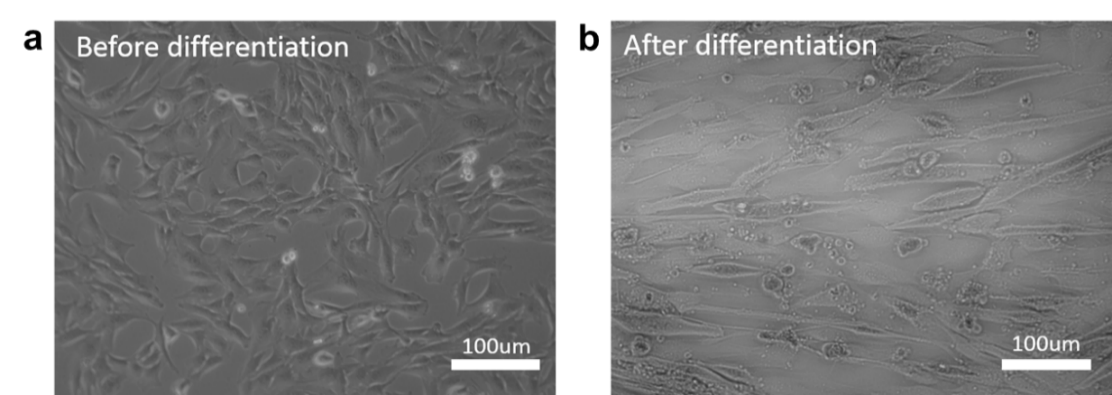


**Fig. S5** The phase contrast images of C2C12 mouse myoblast cells before (a) and after (b) differentiation.

**Table S1**. Average wavelength values with standard deviation for flat (marked as 50%) and uniaxially crumpled (crumpled with 150% and 300% of prestrains) graphene sample. Corresponding plot is shown in blue line in Fig. 1b.

| Prestrains | Wavelength (nm) | Standard deviation (nm) |
| --- | --- | --- |
| 50 | 260 | 42.4 |
| 150 | 154 | 21.6 |
| 300 | 82.7 | 9.16 |

**Table S2.** Average RMS roughness values with standard deviation for flat (marked as 50%) and uniaxially crumpled (crumpled with 150% and 300% of prestrains) graphene sample. Corresponding plot is shown in red line in Fig. 1b.

| Prestrains | RMS roughness (nm) | Standard deviation (nm) |
| --- | --- | --- |
| 50 | 7.49 | 0.975 |
| 150 | 11.8 | 1.62 |
| 300 | 46.3 | 13.3 |

**References**

1. Ou, L. *et al.* Toxicity of graphene-family nanoparticles: a general review of the origins and mechanisms. *Part. Fibre Toxicol.* **13**, 57 (2016).

2. Nezakati, T., Cousins, B. G. & Seifalian, A. M. Toxicology of chemically modified graphene-based materials for medical application. *Arch. Toxicol.* **88**, 1987–2012 (2014).

3. Ema, M., Gamo, M. & Honda, K. A review of toxicity studies on graphene-based nanomaterials in laboratory animals. *Regul. Toxicol. Pharmacol.* **85**, 7–24 (2017).

4. Ku, S. H. & Park, C. B. Myoblast differentiation on graphene oxide. *Biomaterials* **34**, 2017–2023 (2013).

5. Regulatory Data Sheet 3M^TM^ VHB^TM^ Tape 4910. Available at: https://multimedia.3m.com/mws/mediawebserver?mwsId=SSSSSu9n_zu8l00xMx_eP8mxOv70k17zHvu9lxtD7xt1evSSSSSS-. (Accessed: 3rd July 2019)

6. Suzuki, G., Young, R. F. & Suzuki, H. Where are cell-based therapies heading? Current limitations and future directions. *Hear. Res. Open J.* **4**, 71–77 (2017).

7. Mao, A. S. *et al.* Deterministic encapsulation of single cells in thin tunable microgels for niche modelling and therapeutic delivery. *Nat. Mater.* **16**, 236–243 (2017).

8. Bencherif, S. A. *et al.* Injectable cryogel-based whole-cell cancer vaccines. *Nat. Commun.* **6**, 7556 (2015).

9. Yang, H. S. *et al.* Nanopatterned muscle cell patches for enhanced myogenesis and dystrophin expression in a mouse model of muscular dystrophy. *Biomaterials* **35**, 1478–1486 (2014).
